# Toward prevention of stroke

**DOI:** 10.4103/0972-2327.74182

**Published:** 2010

**Authors:** Sanjeev V. Thomas

**Affiliations:** Editor, Annals of Indian Academy of Neurology, Department of Neurology, Sree Chithra Thirunal Institute for Medical Sciences and Technology, Thiruvananthapuram, India. E-mail: sanjeev.v.thomas@gmail.com

World Stroke Day was observed on 29 October in order to focus world attention on stroke and its impact on human life. This year, the theme for World Stroke Day was “One in Six: Act Now.” The Stroke Day proclamation highlights stroke as a preventable and treatable catastrophe. More than four-fifth of all strokes occur in developing countries. The lifetime risk of stroke after 55 years of age is one in five for women and one in six for men. Those with normal blood pressure (<120/80 mmHg) had half the risk of stroke as those who had high blood pressure (≥140/90 mmHg). Research across the world had shown that unhealthy diet, use of tobacco, high blood pressure, dyslipidemia and physical inactivity increase the risk of stroke. Cardiac disorders such as rheumatic heart disease with valvular involvement, atrial fibrillation and other cardiac arrhythmias and infective endocarditis are important preventable causes of stroke in the young adults. Central nervous system infections and undetected hypercoagulable states are also important preventable causes of stroke in underdeveloped countries. Venous strokes such as cerebral venous sinus thrombois are important causes in young women and men in certain situations. Every country needs to develop a national policy to reduce the risk of stroke. The demographic and social characteristics of different communities may demand adaptation of these policies to suit their special situation. The one in six campaign that the world stroke association is promoting should go a long way in this regard. The medical community, particularly the neurologists, needs to take leadership in increasing the stroke awareness in the society. They also have an important role liaison with health care administrators and policy makers to make stroke prevention a health care priority. We need to increase systematic research to explore the causes and mechanism of stroke in this country. No prevention program would succeed without simultaneous attention to manage stroke cases in the community. Several studies have demonstrated that acute care in a comprehensive stroke program reduces the mortality and morbidity and improves the overall outcome. Comprehensives stroke programs with expertise from neurology, neurosurgery, intervention specialists, speech therapy, physiatry and rehabilitation need to be established and maintained. In India, the communication systems, particularly those based on telephone and electronics, are rapidly expanding. It is now possible to access even remote areas of India by these mechanisms. Interdisciplinary efforts to tap these resources to expand the stroke services to remote and difficult-to-access areas need to be strengthened. Electronic and print media can be used more aggressively to promote the concepts of healthy living and stroke prevention in this country. We at the Indian Academy of Neurology and the Annals of Indian Academy of Neurology are committed to strengthen these efforts to bring down the stroke burden.

